# Combined exercise and nutritional rehabilitation in outpatients with incurable cancer: a systematic review

**DOI:** 10.1007/s00520-019-04749-6

**Published:** 2019-04-03

**Authors:** Charlie C. Hall, Jane Cook, Matthew Maddocks, Richard J. E. Skipworth, Marie Fallon, Barry J. Laird

**Affiliations:** 1 St Columba’s Hospice, 15 Boswall Road, Edinburgh, EH5 3RW UK; 2 Institute of Genetics and Molecular Medicine, University of Edinburgh, Edinburgh, UK; 3 Cicely Saunders Institute of Palliative Care, Policy and Rehabilitation, Kings College London, London, UK; 4 Clinical Surgery, Royal Infirmary, Edinburgh, UK

**Keywords:** Rehabilitation, Palliative medicine, Exercise, Nutrition therapy

## Abstract

**Purpose:**

The optimal components for rehabilitation in patients with incurable cancer are unclear. However, principles of exercise and nutrition-based interventions used in cancer cachexia may be applied usefully to this population of cancer patients. This systematic review examines current evidence for rehabilitation combining exercise and nutritional support in patients with incurable cancer.

**Methods:**

MEDLINE, EMBASE and Cochrane databases were searched. Eligible studies included patients with incurable cancer and rehabilitation programmes combining exercise and nutritional interventions. Studies of cancer survivors, curative treatments, reviews, case note reviews, protocols and abstracts were excluded. Grading of Recommendations Assessment, Development and Evaluation (GRADE) criteria were applied to patient-important outcomes.

**Results:**

Of the 2424 search results, 67 abstracts were reviewed and 24 full texts examined. Eight studies (*n* = 685) were included comprising two randomised control trials, three prospective, one exploratory and two secondary analyses. All examined multi-modal outpatient programmes. GRADE analysis revealed moderate evidence (B) for improvements in depression and physical endurance, low-quality evidence (C) for quality of life and fatigue and very low–quality evidence (D) for overall function and nutritional status.

**Conclusion:**

There are limited data for multi-modal rehabilitation programmes combining exercise and nutritional interventions in patients with incurable cancer. However, studies to date report improvements in multiple domains, most notably physical endurance and depression scores. This supports the concept that multi-modal rehabilitation incorporating principles of cachexia management may be appropriate for the wider group of patients with incurable cancer. Further, high-quality studies are needed to define the optimal approach and outcome measures.

## Introduction

Patients with cancer are living longer than ever before; indeed, in many cases, cancer is now considered a chronic disease [1, 2]. While this is clearly a positive development, the consequences of patients living longer with cancer are wide and varied. With longer survival comes an increase in morbidity and increased healthcare costs with associated socio-economic implications [3]. There is a need to take a pro-active approach to this evolving situation and to optimise the overall condition of patients living with cancer, including those with incurable disease [4]. Rehabilitation may be one such way of optimising the function and overall quality of life (QoL) of this patient population.

Rehabilitation is a concept widely embraced by Western medicine for the management of acute and chronic illness and has recently been advocated for patients with incurable cancer, including those receiving treatment with palliative intent [5–7]. Although ‘rehabilitation’ for patients with incurable cancer may seem paradoxical, there is a plausible argument that patients whose overall condition is compromised have the most to gain from appropriately tailored intervention [7]. In patients with advanced disease, rehabilitation aims at improving and/or maintaining function where the effects of the illness or its treatment threaten to cause decline, or to ease the transition toward dependency when functional deterioration is inevitable. Promoting patients’ own interests and social engagement and optimising functional independence are fundamental [7]. It is acknowledged widely that rehabilitation in patients with incurable cancer should be multi-modal and tailored [5, 7, 8] yet, there is a lack of evidence as to the most efficacious components of a rehabilitation programme for this patient population [2].

The emerging principles of optimising physical and nutritional function in patients with cancer cachexia would seem appropriate to be applied to a broader rehabilitation concept in all patients with cancer. Cachexia is defined as ‘an ongoing loss of skeletal muscle mass (with or without fat mass) that cannot be fully reversed by conventional nutritional support and leads to protein breakdown, and resultant loss of muscle mass and functional decline’ [9]. It is common in solid tumours, which account for over 50% of cancer deaths worldwide and affects over half of all patients with advanced cancer [10]. Cachexia adversely affects function and QoL and is an independent predictor of poorer treatment response, side-effect profiles and shorter survival [11–13]. The high prevalence of cachexia in patients with incurable cancer alone means that any rehabilitation intervention for this group should consider key components of cachexia.

Cachexia is characterised by involuntary weight loss and a negative energy balance created by reduced oral intake, alterations in body metabolism and inflammation [10]. Dietary interventions alone are not effective in reversing cancer-related cachexia, [11, 14] due to metabolic alterations including elevated energy expenditure, excess catabolism and inflammation [10], which together prevent muscle anabolism (the ‘anabolic blockade’) [11]. Exercise stimulates skeletal muscle anabolism, leading to increased muscle mass and strength; however, supra-normal protein intake is required to achieve the same post-prandial anabolic effects in cachectic patients [11]. Introducing exercise without nutritional support in this group of patients may exacerbate the negative energy balance. Work to date has demonstrated that cancer cachexia is best targeted through a pro-active, multi-modal intervention that aims to improve lean mass (muscle), physical function and overall QoL [11, 15]. This pro-active and multi-modal approach advocated for cancer cachexia has the potential to be adopted usefully as a rehabilitation approach for patients with incurable cancer.

Patients with incurable cancer frequently suffer from symptom clusters (SCs), where two or more interrelated symptoms present together, independent of other SCs: raising the possibly of a common aetiology or mechanism [16]. Examples include the fatigue/anorexia-cachexia and the fatigue/neuro-psychological clusters, which have been clinically and statistically defined. Proinflammatory cytokines may play a role in the aetiology of SCs [17], and thus, the multi-modal rehabilitation approach advocated for cancer cachexia may also play a useful role in the management of SCs.

Exercise is feasible in patients with incurable cancer and has multiple beneficial effects on physical well-being, fatigue and depression, all impacting on overall QoL [2, 18]. Based on work to date, there is a strong rationale that exercise and nutrition in combination should be key constituents of any rehabilitation programme for patients with cancer; however, the details of any such programme remain to be elucidated [5]. The aim of this systematic review is therefore to examine current evidence for combined exercise and nutritional rehabilitation interventions in patients with incurable cancer.

## Materials and methods

**Fig. 1:**
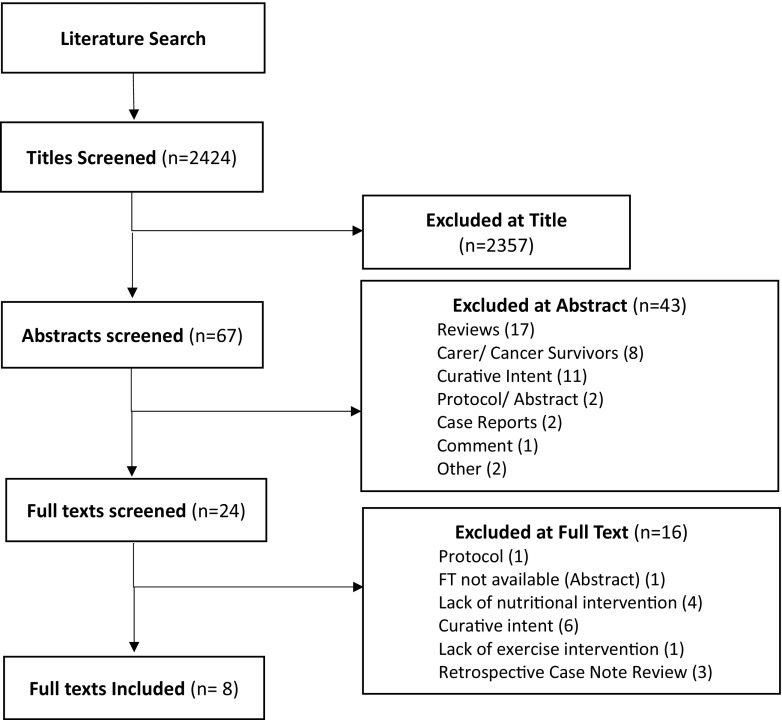
Consort diagram to show the literature search process

Ethical approval was not required for this systematic review. The following databases were searched electronically: MEDLINE, EMBASE and the Cochrane Library. The time frame was 1990 to current. The keywords and search strategy are outlined in Appendix 1. The literature search was performed between February 26, 2018 and March 5, 2018. A consort diagram (Fig. 1) was performed as per PRISMA guidelines.

**Table 1:** Study summaries (in alphabetical order of first author surname; for abbreviations see Glossary below the table)

Author and Year	Design	Participants	Setting	Intervention	Comparator	Aim(s)	Outcome measures (time points)	Main findings and effects (sub-headings relate to the ‘Results’ section)	GRADE
Chasen M. et al. 2010	Observational study	*N* = 53, stage 2–4 gastro-oesophageal cancer	Outpatient clinic (Montreal, Canada)	8-week CNRP	Nil	Evaluate whether an individualised rehabilitation programme affects symptoms and quality of life	-ESAS -PG-SGA -BFI -DT -6MWT (Pre and post)	Physical endurance/depression: Significant improvements in appetite, strength, nervousness, pain, depression, constipation, and nausea. Non-Significant improvement in mean 6MWT distance. Significant reduction in distress. QoL/fatigue: Significant improvements in enjoyment in life, general activity, usual fatigue and fatigue now. Nutritional status: Significant reduction in median PG-SGA scores. Dropout rates: Dropout rate (36%) due to disease progression/ death, (23%) unable to attend regularly enough to be included.	D^a^
Chasen M. et al. 2013	Exploratory study	*N* = 116, stage 3–4 heterogeneous cancers (completed anti-cancer treatments)	Outpatient Clinic (Ottawa, Canada)	8-week PRP	Nil	1. Effect of the PRP on physical, nutritional, social, and psychological functioning. 2. Determine medical factors associated with programme completion.	-ESAS -MD Anderson Symptom Index -PG-SGA -MDFI -BBS -Functional reach test -TUG -Grip test -6MWT -ECOG PS -FBC, serum electrolytes, CRP, alb, TSH, glu, LDH (Pre and post)	Physical endurance/ overall function: Significant improvements in ECOG PS, endurance, mobility, nutrition, general fatigue, and physical fatigue. Moderate non-significant improvement in walking, balance and HGS. Nutrition: Significant improvement in overall nutritional risk. Depression/Fatigue: Small-to-moderate (non-significant) improvements in symptom interference in mood, enjoyment, general activity, and work; decreased activity; balance and function; and several symptoms. Moderate non- significant improvements in: severity of drowsiness, appetite symptoms, interference in relationships and decreased motivation. No worsening of symptoms in any domain. Dropout rate/predictors of completion: 42% did not complete (23% disease progression, 16% personal/ unknown, 2% died, 1% too well). Patients were more likely to complete the programme if CRP was < 10.	C^b^
Feldstain et al. 2016	Secondary analysis of quasi-experimental data	*N* = 131, stage 3–4 heterogeneous cancers	Outpatient clinic (Ottawa Canada)	8-week PRP	Nil	To examine the impact of three aspects of the PRP (inflammation, self-efficacy and exercise), on depression.	-Serum CRP -6MWT -General self-efficacy scale -HADS depression subscale (Pre and post)	Physical endurance: Significant increase in exercise (6MWT). Depression: Significant increase in self-efficacy. Significant decrease in depression scores, but below the 1 clinical level (i.e. none, low, moderate, severe). Predicted variables accounted for 15% change in depression scores. Of the three variables only change in self-efficacy accounted for a significant (11%) change in depression scores. No significant contribution from exercise/CRP. Dropout rate: 39% did not complete the programme (18% disease progression, 18% personal/ unknown, 1% death, 1% geographically inaccessible, 1% active treatment).	D^c^
Feldstain et al. 2017	Secondary analysis of quasi-experimental data	*N* = 44, stage 3–4 heterogeneous cancers (completed anti-cancer treatment)	Outpatient clinic (Ottawa Canada)	8-week PRP	Nil	To ascertain if reductions in depression are maintained 3 months post-PRP completion.	-HADS (T1 *=* Pre-PRP T2 = completion T3 = 3/12 post-PRP)	Depression: Statistically and clinically significant decreases in reported depressive symptomatology between T1, T2 and T3 indicating the PRP helps reduce mild depressive symptomatology and is maintained at 3 months post. Dropout rate: 47/103 (46%) eligible participants included in analysis. Non completers: 14% unreachable and 40% non-responders.	C^d^
Gagnon et al. 2013	Uncontrolled prospective intervention study	*N* = 188, stage 3–4 heterogeneous cancers and haematological cancers not eligible for BMT	Outpatient clinic (Montreal, Canada)	10–12-week CNRP	Nil	To report the degree to which a CNR programme improves symptom control, nutrition status, physical function, psychological well-being, and overall quality of life	-Modified ESAS adapted for palliative patients (QOL and symptom scores) -MDFI -DT -CT -6MWT 5 m walk test -6 month recall weight loss -weight -Presence of alterations of taste/smell. (Pre and post)	Fatigue/weakness/insomnia: Significant reduction in weakness. Small reductions (effect size 0.4) in: sleepiness, insomnia, pain, anorexia. Strong improvements in MDFI activity and physical fatigue (effect size 0.8–1.1). Small improvements in motivation & mental fatigue (effect size 0.4). Depression/QoL: Significant reduction in depression and nervousness. Moderate reduction in distress, coping ability & overall QoL. Physical endurance/strength: Mean 6MWT improved by 41 m (effect size 0.7) and maximal gait speed by 0.15 m/s (effect size 0.6). Patients attended mean 82% scheduled physio sessions. Nutritional status: 77% maintained weight (within 2 kg), or gained > 2 kg: Significant reduction in taste/smell alterations. Dropout rate/predictors of completion: Programme non-completion (30%) associated with poor ECOG PS, CRP >20 mg/L, poor nutrition status and worse anorexia. Non completers: 7% ‘dropout’ 15% disease progression, 9% died.	C^e^
Glare et al. 2011	Prospective study	*N* = 54, heterogeneous cancers (majority lung, colorectal and upper GI) undergoing variable treatments	Outpatient clinic (Sydney, Australia)	8-week CNRP	Nil	1. To demonstrate feasibility of establishing a CNRP in a cancer centre 2. Determine the benefits and outcomes.	-Weight/BMI -Fat %/FFM -PG-SGA -CRP, albumin -GPS -ESAS -KPS -RHGS -6MWT -1 rep max (Follow-up at 1, 2, 3 and 6 months)	Feasibility: 72% recruitment target achieved, >90% patients reported CNRP as important to them. Nutritional status: Baseline nutrition subnormal in 80%: (critical need for dietary intervention in typical patient). Baseline albumin abnormally low in 26%, baseline CRP elevated (>10 mg/L) in 72%. Patients still in the programme at 2 months had lost less weight, were better nourished, fitter & less likely to have elevated CRP than those who had dropped out. Physical endurance/strength: Median 6MWT and RHGS improved by 1/3rd as well as reductions in ESAS symptom scores. Dropout rate/predictors of completion: High attrition rate noted: 2-month compliance 58%, 44% at 3 months, and 12% at 6 months. Predictors of completion: 6MWT > 420 m and those continuing anti-cancer treatment.	D^f^
Jones et al. 2013	Two-arm randomised (wait list) control trial	*N* = 41, patients at end of treatment or with active, progressive, recurrent haematological or breast cancers. Recruited from Oncology services.	Outpatient hospice day therapies unit (London, UK)	3 -month rehabilitation programme: core components: outpatient clinic, nurse led clinic, day suite, volunteer support and relaxation groups. Other interventions dependent on needs/goals.	Usual care (offered intervention after 3 months)	To test the clinical and cost-effectiveness of the rehabilitation intervention examining: 1. Psychological subscale of the supportive care needs survey long form 2. Other SCNS domains, psychological status, continuity of care and EQ-5D. Economic evaluation based on EQ-5D score	-SCNS-LF59 -K10 -Continuity of care -EQ-5D/ EQ-VAS -Cost-effectiveness analysis: EQ-5D utility values converted to QALYs (Pre and post)	Care needs/health state: Significant differences in physical and patient care subscales of the SCNS and self-reported health state. Other secondary outcomes non-significantly lower in intervention arm. Depression: Significantly lower unmet needs for psychological support for patients receiving the intervention. Cost-effectiveness: Significant reduction in healthcare resource use and a corresponding improvement in QoL Intervention associated with greater total costs and greater QoL (mean difference 0.05 QALYs) resulting in an ICER of £19,391 per QALY gained: cost-effective in 55.4% & 73.3% of simulations at cost thresholds £20,000/ £30,000 respectively. Qol: Effects on sexuality support needs, continuity of care and health related QoL less apparent. Feasibility/dropout rates: Recruitment poor with 41 consented of 81 approached (target 240). 12% did not complete follow-up.	B^g^
Uster et al. 2017	Parallel group randomised control trial	*N* = 58, metastatic or locally advanced GI or lung cancers	Cancer centre (Winterthur, Switzerland)	3-month nutrition and physical exercise programme	Standard cancer centre medical therapy	To test the effects of the programme in terms of 1. Global health status/QoL Scale 2. Dietary intake	-EORTC QLQ-C30 -3-day food diary -HGS -6MWT -30 s sit to stand test -1 Rep max leg press -BIA -Weight -Unexpected hospital days -ECOG PS (measurements pre, 3 months and 6 months)	QoL: No significant difference in global QOL between groups. Nutritional status: Less increase in nausea and vomiting in intervention group compared to control group. No other functional or symptom scale differences seen. Significant increase between groups in daily protein intake but after 6 months this had decreased in both groups to below baseline values. Body weight increased in both groups. Physical endurance/strength/ overall function: All physical parameters improved in intervention vs control group but not to statistical significance. Change in ECOG PS not reported. Feasibility/adverse events: All patients managed at least half a unit of the ONS after training sessions and attended a mean of 3 nutritional counselling sessions. Mean adherence to bi-weekly training sessions 67% and lower dropout rate in intervention group indicating the feasibility of the programme. No adverse effects noted. No significant difference in unexpected hospital stays. No significant difference in survival rates. 58 patients recruited (target 74). Trial cut short due to slow accrual. 63% eligible patients refused to participate.	B^h^

Glossary of Terms: *BBS*, Berg Balance Scale; *BIA*, bioelectrical impedance analysis; *BFI*, brief fatigue inventory; *BMI*, body mass index; *BMT*, bone marrow transplant; *CRP*, C-reactive protein; *CT*: coping thermometer; *DT*, distress thermometer; *ECOG PS*, Eastern Cooperative Oncology Group Performance Status; *EORTC QLQ-C30*, self-reported questionnaire designed to assess quality of life of cancer patients; *EQ-5D/EQ-VAS*, EuroQol-5 Dimensions/Comprising 0–100 Visual Analogue Scale of perceived health state; *ESAS*, the Edmonton Symptom Assessment Scale; *FBC*, full blood count; *FFM*, fat free mass; *GPS*, the Glasgow Prognostic Score; *H&N-35*, Head and Neck Specific EORTC Self-reported Questionnaire with sections relating to head and neck cancer symptoms/issues; *HADS*, Hospital Anxiety and Depression Scale; *ICER*, Incremental Cost-Effectiveness Ratio; *K10*, Kessler Psychological distress scale; *KPS*, the Karnofsky Performance Status; *LDH*, lactate dehydrogenase; *MDFI*, Multidimensional Fatigue Inventory; *PG-SGA*, Patient-Generated Subjective Global Assessment; *QALY*, quality-adjusted life year; *RHGS*, right hand grip strength; *SCNS-LF59*, supportive care needs survey long form; *SOB*, shortness of breath; *TSH*, thyroid stimulating hormone; *TUG*, timed up and go test; *6MWT*, 6-minute walk test

^a–f^All started as GRADE ‘C’ (‘low’) evidence quality due to study type

^g, h^Started as ‘A’ (high) evidence quality due to study type

^a^GRADE score reduced (−1) due to high dropout rate (58% dropout rate), variable intervention, small sample size, small numbers included in analysis. Although effect consistent with rapid effect, GRADE score not increased due to these limitations

^b^GRADE score reduced (−1) due to high dropout rate (%), incomplete analysis of enrolled patients, variable intervention. GRADE score increased (+1) due to magnitude of effect and rapidity across subjects with larger sample

^c^GRADE score reduced (− 1) due to dropout rate (39%), missing data (unquantified), variable interventions in relation to the primary outcome, surrogate outcome measure (HADS) with limited diagnostic sensitivity for clinical vs. subclinical depression. GRADE score not increased due to these limitations

^d^GRADE score reduced (− 1) due to large loss to follow-up and small numbers of participants, sample bias and variable interventions given. GRADE score increased (+ 1) due to rapidity and consistency of effect as well due to attempts to analyse demographic of non-responders (confounding)

^e^GRADE score reduced (− 1) due to use of non-validated tools, variable interventions, unexplained absence of data for outcomes. GRADE score increased (+ 1) due to large magnitude and consistency of effect which was rapid

^f^GRADE score reduced (− 1) due to high dropout rate, variable intervention, lack of adequate control for confounding (67% on chemotherapy), small sample size and missing data. GRADE score not increased due to these limitations

^g^GRADE score reduced (− 1) due to low numbers (17% predicted recruitment), variability of interventions, wide confidence intervals (due to small sample size). GRADE score not increased due to these limitations

^h^GRADE score reduced (− 1) for selection bias and failure to adequately control for confounding and small sample size. GRADE score not increased due to these limitations

### Eligibility criteria

Studies met the following inclusion criteria: patients with incurable cancer (defined as metastatic cancer [histological, cytological or radiological evidence] or locally advanced cancer being treated with palliative intent); rehabilitation programmes including both exercise and nutritional components; all methodologies; studies in humans; and English language.

Studies were excluded if they met any of the following criteria: studies of cancer survivors or carers of cancer patients; unimodal rehabilitation interventions; reviews; protocols; case reports; retrospective case note reviews; conference abstracts; and rehabilitation/prehabilitation for cancers managed with curative intent.

### Appraisal process

Titles were reviewed by CH then relevant abstracts screened by CH and BL. Abstracts deemed relevant or requiring clarification were reviewed at full text. Full texts were screened by CH and BL and thematic analysis applied by JC and CH. Estimates of effect extracted from studies included change scores (pre-post measurements), effect sizes and *P* values. Values were synthesised according to patient-important outcomes (see below) as well as outcomes of methodological interest for future study design: feasibility, dropout rates, predictors of completion and cost-effectiveness.

Grading of Recommendations Assessment, Development and Evaluation (GRADE) analyses were undertaken by CH and JC. Due to the complexity and to improve inter-rater reliability, a checklist was developed [Supplementary material-on request] based on the GRADE handbook and a validated checklist for meta-analyses [19–21]. This was applied to individual studies then to the body of evidence for patient-important outcomes, which were decided a priori between authors and ranked in order of importance. Where GRADE discrepancies existed, these were discussed among the authors and a consensus reached.

## Results

Figure 1 shows the literature review process. The following numbers of articles were retrieved from each database: 781 (MEDLINE), 1625 (EMBASE) and 18 (Cochrane Database of Systematic Reviews).

A summary of the included studies is detailed in Table 1. Eight studies were eligible enrolling a total of 685 participants. Studies included two randomised control trials (RCTs) [22, 23], three prospective studies [24–26], two secondary analyses of quasi-experimental data [1, 27] and one exploratory study [28]. All interventions were outpatient-based rehabilitation programmes: seven in hospitals and one hospice-based. Three studies examined the 8 to 12-week McGill Cancer Nutrition Rehabilitation Programme (CNRP) [24–26], and three studies examined the 8-week-Ottawa Palliative Rehabilitation Programme (PRP) [1, 27]. Two studies examined novel rehabilitation programmes in the UK [23] and Switzerland [22]. All programmes were interdisciplinary and were individually tailored. Seven studies included core components combining dietary modification/supplementation and exercise [1, 22, 24–28]. The remaining study included dietary and physiotherapy interventions as an optional (non-core) element dependent on patient goals: it was not possible to ascertain numbers of participants receiving input from both these specialists [23].

Studies and patient-important outcomes and were evaluated using the GRADE approach. Consensus was reached on the quality of evidence for each patient-important outcome, presented in Tables 2 and 3.

**Table 2:** Summary of findings: modified due to study types. Patients or population: patients with incurable cancer. Settings: outpatient. Intervention: multi-modal rehabilitation programmes comprising exercise and nutritional elements. Comparison: where available-standard care

Patient- important outcomes	Studies	*N* = total participants* (breakdown per outcome measure)	Quality of the body of evidence (GRADE)	Comments
Quality of life	3 [22, 23, 26]	*N* = 214 129 (ESAS) 41 (EQ-VAS) 44 (EORTC C30)	Low (C)	Two moderate quality studies with conflicting results, one low-quality study showing improvement, studies have limitations and inconsistencies in outcome variables.
Overall function	2 [25, 28]	*N* = 81 56 (ECOG PS) 25 (KPS)	Very low (D)	Two studies with low and very low-quality examined changes in functional status scores, one finding significant and one non-significant improvements. Sparse data with limitations.
Fatigue	4 [22, 24, 26, 28]	*N* = 203 22 (BFI) 137 (MDFI) 44 (EORTC QLQ-C30)	Low (C)	Two low, one very low-quality studies with limitations showing significant improvements in fatigue in spite of sparse data, and one high-quality (underpowered) study showing non-significant improvements in intervention group compared to control
Physical endurance/strength	6 [22, 24–28]	*N* = 342 6MWT (342) HGS (64 within two of the above studies)	Moderate (B)	Six studies with quality overall low quality, with limitations: variable consistency in significance levels but overall magnitude of effect seen was improvement in spite of low statistical power of studies: GRADE of evidence increased (+2)
Depression	6 [1, 23, 24, 26–28]	***N*** **=** 371 211 (ESAS) 124 (HADS) 36 (SCNS-LF59)	Moderate (B)	Overall low-quality studies with limitations but GRADE of evidence increased (+2) due to studies all showing consistent significant improvements in depression/psychological subscales.
Nutrition/weight	5 [24, 26, 28]	*N* = 285 107 (PG-SGA) 178 (weight)	Very low (D)	Five studies of overall low quality with serious limitations and indirectness (variable interventions). Two low-quality/very low–quality studies showed improved PG-SGA scores but the highest quality RCT showed only significant increases in protein intake. Evidence not strong enough to be upgraded.

*Total participants include numbers actually analysed within studies for each outcome as opposed to Table 1 showing ‘*N*’ as numbers enrolled into each trial

**Table 3:** GRADE Definifions

GRADE	Definition (from [20])
High (A)	We are very confident that the true effect lies close to that of the estimate of the effect.
Moderate (B)	We are moderately confident in the effect estimate: the true effect is likely to be close to the estimate of the effect, but there is a possibility that it is substantially different
Low (C)	Our confidence in the effect estimate is limited: the true effect may be substantially different from the estimate of the effect.
Very low (D)	We have very little confidence in the effect estimate: the true effect is likely to be substantially different from the estimate of effect

### Feasibility and adverse events

Three studies (*n* = 300) commented on feasibility of the rehabilitation programmes or constituents of their interventions. Patients attended a mean of 67% of bi-weekly exercise training classes over 3 months, and all patients managed at least half of the ONS after each training session in one RCT [22]. Similarly, for a 10–12-week CNRP, patients attended 82% of prescribed exercise sessions [26]. No adverse effects were reported, but this was only mentioned in one study [22]. In the same trial, 3-month dropout rates due to death or withdrawal were lower in the intervention group compared to the control group: 4% vs. 24%, indicating feasibility. Over 90% of patients reported the CNRP as important to them; however, introducing this programme in a busy cancer centre was labour-intensive, requiring a nurse, administrative and financial support to be viable [25].

### Dropout rates and predictors of programme completion

Completion rates from CNRP/PRP programmes ranged from 42 to 70% [24, 26]. Dropout rates due to disease progression/death accounted for between 49 and 61% [27, 28]. Other reasons included geographical inaccessibility (39%) [24] or unknown/personal reasons (37%) [28]. Schedules of medical appointments made it hard to adhere to the CNRP, and at times, the amount of information to take in could be overwhelming [25]. Predictors of programme completion included lower baseline CRP levels [24, 26], lower ECOG performance status and better nutritional status [26]. Glare et al. [25] cited a baseline 6-min walk test (6MWT) > 420 m, (i.e. better endurance) as a predictor of programme completion. Although within this study programme completers demonstrated improvements in multiple domains, high dropout rates (> 50%) meant that earlier identification of the population who would best respond was recommended.

### Physical endurance, strength and overall function

Studies used multiple outcome measures; however, the 6MWT was frequently cited as a marker of endurance and mean distances improved in six studies (*n* = 342). Two studies reported performance status (ECOG/KPS) as primary endpoints (*n* = 81).

Feldstain and Chasen [27, 28] reported significant increases in mean 6MWT distance (*t*(79) = − 3.91, *P* = < 0.001 [27] and *d =* 0.80, i.e*.* moderate-to-large effect size, *P* < 0.001 [28]) after the PRP. Studies utilising the CNRP quoted improvements in mean 6MWT distances between 41 m (95% CI 29–52 m: effect size 0.7, *P* not reported [26]) and 58 m [24] (non-significant, median 6MWT increase: *P* = 0.01). Glare and Uster [22, 25] reported non-significant increases in 6MWT (*n* = 25, median 441 m (186–675) to 570 m (range not reported) [25], data presented graphically [22]) and other physical parameters, though both studies were underpowered. Chasen [28] reported an improvement in ECOG PS (*P* < 0.001, *t* = 6.43, *d* = 0.90) from mean 1.8 (± 0.7) to 1.29 (± 0.46) for patients completing the PRP, and Glare [25] reported non-significant improvements in median KPS score (*n* = 25) from 70% (score ≥ 50%:100%) to 80% (score ≥ 50%:100%) in programme completers.

### Fatigue, weakness and insomnia

Four studies described changes in fatigue (*n* = 211) using the Brief Fatigue Inventory (BFI) [24], the Multidimensional Fatigue Inventory (MDFI) [26, 28] and the European Organisation for Research and Treatment of Cancer (EORTC QLQ-C30) symptom scales [22].

Chasen [24] described improvements in BFI usual fatigue (5.0 (1–10)–3.0 (1–9); *P* = 0.03) and fatigue now (5.0 (0–10)–3.0 (0–10); *P* = 0.05). Furthermore, in 2013, using the MDFI, reductions in general and physical fatigue (*d* = 0.61 and 0.55, both *P* < 0.001) were reported [28]. Gagnon reported strong improvements in MDFI activity and physical fatigue (mean 4.6 [95% CI 3.6–5.6] to 3.7 [95% CI 2.6–4.7] respectively, both *P* < 0.001, effect size 0.8–1.1), moderate reductions in general fatigue (mean change 2.8 [95% CI 1.8–3.8], *P* < 0.0001, effect size 0.7) and small but significant improvements in motivation and mental fatigue (mean change 1.6 [95% CI 0.8–2.5], *P* = 0.0004 and 1.7 [95% CI 0.8–2.6] *P* = 0.0005: effect size both 0.4). Reductions were seen in weakness (mean change 1.5 [95% CI 1.1–1.8], *P* < 0.0001, effect size 0.7) as well as reductions in sleepiness and insomnia (mean change 1.1 [95% CI 0.6–1.6], *P* < 0.0001 and mean change 1.0 [95% CI 0.5–1.4], *P* = 0.0001 effect size both 0.4) [26].

### Effects on depression and quality of life

Six studies included endpoints examining depression (*n* = 371) using the Edmonton Symptom Assessment Scale (ESAS) [24, 26, 28], the Hospital Anxiety and Depression Scale (HADs) [1, 27] and the psychological subscale of the Supportive Care Needs Survey Long Form (SCNS-LF59) [23]. Studies frequently mentioned QoL but only three studies reported a QoL outcome using questions from the ESAS [26], EORTC QLQ-C30 [22] and EQ-5D/EQ-VAS questionnaires [23].

Chasen reported improvements in (ESAS) nervousness and depression (4.5 (0–10)–1.5 (0–5); *P* = 0.02 and (3.0 (0–9)–2.0 (0–7); *P* = 0.04 respectively) in 2010 [24] and depression scores for those completing the PRP in 2013 (*P* = 0.005, *d* = 0.37) [28]. Similarly, Gagnon [26] reported reductions in (ESAS) depression scores (mean change 1.4 (95% CI 1.1–1.8) *P* < 0.0001, effect size 0.7) as well as reduced (DT) distress (mean change 1.4 (95% CI 0.9–1.9) *P* < 0.0001, effect size 0.5), improved (CT) coping (mean change 1.8 (95% CI 1.2–2.4) *P* < 0.0001, effect size 0.7) and (ESAS) QoL (mean change 1.0 (95% CI 0.6–1.3) *P* < 0.0001 effect size 0.5) after the CNRP. One RCT demonstrated reduced unmet psychological support needs on the psychological subscale of the SCNS compared with controls (adjusted difference − 16.8 points (95% CI − 28.34 to − 5.3) *P* = 0.006) and improvements in (EQ-5D) self-reported health state (12.8, (95% CI 3.2–22.4) *P* = 0.01) [23]. Conversely, the other RCT [22] showed no difference global QoL. There was a non-significant trend toward improvement; however, this trial was curtailed due to poor recruitment and lacked power. Feldstain [27] described increased self-efficacy (27.86 (SD = 6.16) to 31.23 units (SD = 5.77), *P* < 0.001) and reduced depression scores (7.14 (SD = 3.91) to 5.95 units (SD = 3.51), *P* = 0.002) after the PRP. Changes in ‘self-efficacy’ (the perception that one can influence life events/quality of functioning) accounted for the greatest change (11%) in depression scores. In a subsequent study [1], depression score improvements were maintained 3 months post-PRP (mean difference T1–T3 = 2.21, SE 0.78, *P* = 0.007).

### Nutritional status

Two studies measured weight as an outcome [22, 26], two used the Patient-Generated Subjective Global Assessment (PG-SGA) [24, 28] and one used a combination of both [25]. Comparison between studies is hampered by lack of detail on nutritional interventions, heterogeneity of subjects and varied outcome measures. Nutritional counselling, dietary advice and oral nutritional supplements (ONS) are mentioned by most. Details of dietary interventions varied: 72% saw the physician, physiotherapist and dietitian, with 25% seeing the physician and dietitian only in one [25]; 60–70% saw the dietitian in another [1]; and in another, 94.7% received dietary counselling, with 80.2% receiving ONS [26]. One RCT ensured patients received > 1.2 g protein/kg/day and encouraged protein dense ONS (18–20 g in 125–200 mL) after exercise. Significant improvements in protein intake (*P* = 0.01), but no significant differences in energy intake or nutritional status were seen between arms: indeed, weight increased in both [22]. Patients undergoing nutritional interventions within multidisciplinary programmes maintained (77% within 2 kg) [26] or increased their weight [22], although longitudinal data is lacking. Increases in protein intake were not maintained 3 months post-intervention, dropping below baseline in both groups, more so in the control group [22].

PG-SGA score improvements (median baseline 12.0 (2–24), to 9.0 (1–18) at completion *P* = 0.05) were reported following the CNRP [24] and also post-PRP (baseline mean (± SD) 8.15 (± 5.29) to 5.98 (± 4.14), *t* = 3.49, *P* = 0.001, *d* = 0.46) [28]. There was a higher mean PG-SGA score (89% ≥ 9 versus 70% ≥ 9) in dropouts of than those who returned for their 2-month CNRP follow-up [25].

### Cost-effectiveness

One RCT (*n* = 41) examined the cost-effectiveness of a 3-month, complex hospice-based rehabilitation programme plus usual care versus usual care alone [23]. The intervention was associated with greater total costs (mean difference £955, 95% CI £82–£1975) and greater QoL (mean difference 0.05 QALYs, 95% CI 0.000–0.112) resulting in an incremental cost-effectiveness ratio (ICER) of £19,391 per quality-adjusted life year (QALY) gained. The cost per QALY was only calculated over the 3-month (intervention) period and was close to the £20,000 threshold often used for incorporation of an intervention into the UK National Health Service. The authors postulated that if the benefits of the programme were maintained for 1 year, the ICER would decrease to approximately £4400 making the projection cost-effective in 92.7% of simulations at a threshold of £20,000 per QALY.

## Discussion

There are few data available for multi-modal rehabilitation programmes incorporating exercise and nutritional interventions for patients with incurable cancer. However, of those outcomes important to patients, many showed improvements following the interventions described. Factors associated with programme completion are higher baseline nutritional or functional status and lower levels of inflammation. Of the studies analysed, methodological quality was frequently limited by study design and statistical power. Heterogeneity of study design (including interventions and outcome measures) meant meta-analysis was not appropriate.

In patients with incurable cancer, the highest quality of evidence pertains to improvements in depression and physical endurance following multi-modal rehabilitation programmes including exercise and nutritional support. Depression is one of the commonest mental health problems in patients with advanced cancer [29]. Six studies showed improvements in depression scores, using outcomes including the Hospital Anxiety and Depression Scale (HADs). This scale, however, does not differentiate clinical depression from sub-threshold symptomatology, which is a limitation to its use in this patient population [27].

A high level of evidence exists for exercise in rehabilitation trials [2, 30], and this review suggests that the combination of exercise and nutritional support also improves physical endurance in patients with incurable cancer. Evidence for change in overall function remains very low due to serious limitations in the evidence (Table 2). Plausibly however, improvements in physical endurance may impact on overall function via reductions in dependency.

Evidence for improved fatigue remains low. This finding is in keeping with the lack of interventions for fatigue in advanced incurable disease. Rehabilitation studies in patients with cancer are at risk of selection bias as patients recruited may be more motivated, acknowledged by Uster [22]. Three studies measured QoL, but overall evidence for improvement remained low. Cancer negatively affects QoL by many modalities; hence, the necessity of a multi-modal approach in this patient group. Results for nutritional parameters were variable, and it was difficult to make comparisons, resulting in a very low rating of evidence. Weight is a key feature of cachexia and (as an outcome) is meaningful to both patients and clinicians [31], but does not take into account body composition. PG-SGA scores reflect changes in weight but also symptoms so may not reflect alterations in nutritional status alone. Furthermore, patients with incurable cancer are more likely to be at a ‘refractory’ stage of cachexia that is poorly responsive to treatment; therefore, this level of evidence is unsurprising [9]. A further confounding factor is that of contamination, whereby the control group mimics the intervention. Both groups gained weight and improved hand grip strength within Uster’s RCT, which may have contributed to a lack of statistical significance [22].

Cancer rehabilitation trials are frequently limited by design and sample size and high attrition rates are common [32]. Recruitment issues were encountered in both RCTs; one cut short due to poor recruitment [22], the other recruiting just 17% of expected patients. In this RCT, 189 eligible patients were not approached, and interviews with recruiting clinicians revealed reasons including discomfort with the trial design, lack of confidence discussing prognosis and anxieties about delivering the intervention at a hospice [23]. Other barriers to recruitment include difficulties identifying participants (complex inclusion criteria) and high refusal rates (competing priorities, fear of randomisation to non-preferred arm, lack of acceptable control). Healthcare professional gatekeeping is one of the most significant barriers to recruitment [33]; however, patients find symptom control trials beneficial irrespective of whether they obtain improvements in their symptoms [34].

Some of the findings presented herein are worthy of comparison to other diseases. The importance of exercise and nutritional intervention is acknowledged in models of rehabilitation for non-malignant disease, where cachexia may be present. Pulmonary rehabilitation (PR) has included exercise as a cornerstone for many years. Research on muscle dysfunction in patients with chronic obstructive pulmonary disease (COPD) has shown that multi-modal interventions including exercise and nutritional supplementation can have beneficial effects on body weight, exercise tolerance, physical activity, depression and survival [35, 36]. There is now a shift toward earlier PR to improve exercise tolerance and physical activity and to promote self-efficacy and behavioural change while reducing exacerbations [36]. These observations provide further grounds for optimism that exercise and nutrition-based rehabilitation programmes in patients with incurable cancer are viable.

It is clear from work to date that the principles employed in the treatment of cancer cachexia may be useful in rehabilitation. Work is ongoing to define the best approach to target cachexia at all stages of disease: including ‘prehabilitation’ for patients undergoing cancer surgery [37], and a phase 3 trial is underway of a multi-modal cachexia treatment (exercise, nutrition plus anti-inflammatories) for patients undergoing chemotherapy [31]. A feasibility trial of a multi-modal rehabilitation programme combining exercise and nutritional support for hospice outpatients with incurable cancer is also in progress [38]. There is a growing body of evidence for the use of new technologies in oncology trials such as physical activity monitors, which provide an objective measurement of patient activity in their usual environment [39]. There is now strong international consensus that cachexia is a multi-modal problem which requires multi-modal treatment [10, 11]. One of the challenges in cancer cachexia, however, is that the optimal endpoints are not clear, and this appears similar in cancer rehabilitation studies where consensus on endpoints is not evident [40]. Potential outcomes are numerous, though it is important that measures are validated and clinically meaningful [30]. GRADE discourages the use of ‘surrogate outcomes’, which can result in downgrading of evidence for indirectness [20]. The aforementioned difficulties in comparing trials due to the clinical and methodological heterogeneity of interventions and outcomes may be one reason for the slow growth of evidence in this field. There are inherent difficulties however, performing clinical trials in a field where personalised care makes standardising interventions challenging [30].

For patients with incurable cancer, concerns about nutrition, loss of function and increased dependency are commonplace. Loss of independence can compromise a person’s sense of dignity and fears of functional decline can surpass fears of impending death [41]. As the population changes, with improvements in anti-cancer treatment and greater numbers of patients treated under the umbrella of palliative care, there is the need to enable patients to live their lives as fully as possible, while minimising social-care costs. This approach, incorporating rehabilitation, places living before dying and is at the heart of palliative care [6].

Limitations

The search strategy may have precluded relevant articles due to stringency of the search criteria. One such factor was exclusion of studies reporting results for ‘cancer survivors’. The definition of this term is very broad, encompassing patients from initial diagnosis to death, and may also include family, friends or caregivers [42]. Application of the GRADE criteria can be advantageous due to transparency of judgements about quality; however, limitations of the system (including its use for assessment of individual studies) are acknowledged [43]. A further challenge with GRADE is the complexity which can result in poor-to moderate inter-rater agreement [44]. Our GRADE checklist was designed to improve this and, though effective, it is not a validated tool. The lack of randomised control trials (two studies) meant that meta-analysis was not possible. However, the use of the robustly validated GRADE system of analysis [19–21] ensured that conclusions drawn were as accurate as possible.

## Conclusion

This review demonstrates that in spite of limited data, multi-modal rehabilitation programmes incorporating exercise and nutritional interventions improve many outcomes that are important to patients with incurable cancer, most notably those relating to physical endurance and depression. This finding, along with factors associated with programme completion, lends further support to the argument that exercise and nutritional intervention should form integral components of cancer rehabilitation. Multi-modal treatments are evolving for cancer cachexia, and these may be usefully adapted to cancer rehabilitation.

There are multiple opportunities to improve patient well-being throughout all phases of cancer care: from the point of diagnosis, prior to treatment and at the advanced stages of incurable disease [4, 8]. Modern palliative care should now encompass rehabilitation [6] as well as forming an integral and concurrent element of active cancer care [45]. Rehabilitation for patients with incurable cancer has the potential to significantly improve functional status and QoL for the ever-increasing numbers of patients ‘living with cancer’, with potentially large socio-economic benefits. Further, carefully designed high-quality trials are needed, but the current shift toward a joint rehabilitative-palliative approach throughout the cancer trajectory shines a light in the dark for cancer patients of the future.

## Appendix. Search Strategy

All terms searched within ‘Title’. Limits: Human subjects, English language, year 1990–current.

MEDLINE. Total 781

1. Rehabilitation + Cancer: (578)
2. Rehabilitation + Cancer + Exercise: (36)
3. Rehabilitation + Cancer + Nutrition: (3)
4. Rehabilitation + Cancer + Exercise + Nutrition: (0)
5. Exercise + Cancer + Nutrition: (21)
6. Palliative + Rehabilitation: (42)
7. Palliative + Exercise + Nutrition: (0)
8. Prehabilitation: (81)
9. Prehabilitation + Cancer: (18)
10. Prehabilitation + Cancer + Palliative: (0)
11. Prehabilitation + Palliative: (0)
12. Prehabilitation + Nutrition (2)

EMBASE. Total 1625

1. Rehabilitation + Cancer: (1168)
2. Rehabilitation + Cancer + Exercise: (65)
3. Rehabilitation + Cancer + Nutrition: (10)
4. Rehabilitation + Cancer + Exercise + Nutrition: (0)
5. Exercise + Cancer + Nutrition: (50)
6. Palliative + Rehabilitation: (96)
7. Palliative + Exercise + Nutrition: (3)
8. Prehabilitation: (180)
9. Prehabilitation + Cancer: (50)
10. Prehabilitation + Cancer + Palliative: (0)
11. Prehabilitation + Palliative: (0)
12. Prehabilitation + Nutrition (3)
  Cochrane Library. Total 18
13. Rehabilitation + Cancer: (2)
14. Rehabilitation + Cancer + Exercise: (4)
15. Rehabilitation + Cancer + Nutrition: (0)
16. Rehabilitation + Cancer + Exercise + Nutrition: (0)
17. Exercise + Cancer + Nutrition: (1)
18. Palliative + Rehabilitation: (2)
19. Palliative + exercise + nutrition: (0)
20. Prehabilitation: (1)
21. Prehabilitation + Cancer: (8)
22. Prehabilitation + Cancer + Palliative: (0)
23. Prehabilitation + Palliative: (0)
24. Prehabilitation + nutrition: (0)
